# Notes and News

**Published:** 1898-01-08

**Authors:** 


### Notes and News.

(Collected and Communicated.)

The Chair of Medical Jurisprudence is vacant at the University of Glasgow, owing to the retirement of Professor Simpson through ill-health.

The Hampton District Council resolved to adopt the new bacteriological process at its sewage works, and to reconstruct them accordingly.

The Queen has consented to become patron of the Royal British Institute of Public Health, and the office of the Institute has been removed to 197, High Holborn.

Mr. Noble Smith lectured at the City Orthopædic Hospital on January 4th, at half-past five, on "Wry Neck and some Other Contractions."

Miss Beatrice A. Sharp, matron of East Cornwall Hospital, Bodmin, Cornwall, has been awarded £10 by the Board of Management in recognition of her services to that establishment.

At the second annual meeting of the Elgin District Nursing Association it was resolved to appoint a second nurse. The additional cost will be £80 per annum, but the result will be to make the working of the association still more satisfactory.

Italy has sustained a great loss through the death of the Senator, Francesco Brioschi. He founded the Polytechnic Institute at Milan and was president of the Accademia dei Lincei. He fell a victim to duty, having contracted malarial fever whilst superintending some important drainage works.

The Great Roll of Ministering Children is growing apace. The children of T.R.H. the Duke and Duchess of York were photographed at Sandringham last week. Messrs. Speaight, 178, Regent Street, have been commanded to Osborne on Tuesday next to photograph the Royal children of the Duke of Connaught and Princess Beatrice's children. We hope that our readers who have children will encourage them to follow the example of the Royal children.

An interesting festivity took place at Acton on New Year's Day. At Friars Place there is a home of rest, where horses past work are received and cared for to the end of their days. At this institution the twenty-nine inhabitants were regaled with all the dainties known to be dear to the equine heart. Each horse had a large supply of apples, carrots, and bread delicately seasoned with sugar. The oldest inhabitant is a mare of 41 years of age. A famous charger, called "Bones," once belonging to the Horse Guards, was amongst the number who feasted on New Year's Day.

Sir William Gowers has been elected an honorary Fellow of the Royal College of Physicians of Ireland.

Mr. E. Carbery, M.B., B.Ch., B.A.O., Dublin, has been appointed House Physician to the City of London Hospital for Diseases of the Chest.

We are asked to state that radiographs and objects of interest will be shown at each meeting of the Röntgen Society. The next meeting takes place on January 8th, at 11, Chandos Street, Cavendish Square.

The *Medical Week*, which was a sort of English edition of the *Semaine Medicale*, will no longer be published. Dr. Gubb, who did the English side, goes to the *Medical Press and Circular*.

We have also to record the decease of another little paper—the *Guyoscope*—which, although not medical, was for "medicals." It was funny and was clever, so we suppose it fulfilled its mission; and if it irritated some people—well, perhaps that was its mission too.

In regard to an article in The Hospital of December 25th on "Milk Laboratories," we are informed that the Friern Manor Dairy Farm (Limited), the head office of which is at 20, Farringdon Street, E.C., are prepared to dispense milk prescriptions after the method of Dr. Rotch, which we described.

Amongst the New Year honours, the following have been awarded to members of the medical profession: Dr. John Battynike and Dr. John Struthers, Professor of Anatomy, Aberdeen, have been knighted; and Dr. Gairdner, Professor of Medicine at Glasgow and Physician-in-Ordinary to the Queen, has been created a Knight Commander of the Bath.

Sir John Hutton delivered an address before the Sanitary Inspectors' Association, of which he is president, on New Year's Day. In alluding to the improved sanitation of London, he expressed the belief that the death-rate would be still further reduced in the future, and expressed the hope that greater attention would be paid to securing an unimpeachable water supply forthwith.

The Prince of Wales's Hospital Fund for London has received from the East and West Marylebone Committee (Sir Horace Farquhar, Bart., M.P., chairman) a further payment of £1,339 3s. 9d., making a total of £1,599 8s. 9d.; from the Court of Assistants and Members of the Honourable Artillery Company £244 8s. 8d.; and from the Great Eastern Railway Company, contributed by the staff through the weekly and monthly payments, £140.

When so many classes of the community have received ambulance instruction it is strange that nothing in this direction has been instituted amongst our police force. In all scenes of accident the police-constable is an almost invariable attendant, and many lives would be saved and further injuries averted by a small amount of knowledge of first aid on the part of the police. Quite recently a policeman, possessing knowledge lacking amongst his craft in general, was the means of restoring to life a man who had hung himself in a shed at Braintree. The policeman resorted to artificial respiration, with the result that the man breathed normally in forty minutes.

